# The Last Mile: Where Artificial Intelligence Meets Reality

**DOI:** 10.2196/16323

**Published:** 2019-11-08

**Authors:** Enrico Coiera

**Affiliations:** 1 Australian Institute of Health Inovation Macquarie University Sydney Australia

**Keywords:** artificial intelligence, implementation sceince, sociotechnical systems

## Abstract

Although much effort is focused on improving the technical performance of artificial intelligence, there are compelling reasons to focus more on the implementation of this technology class to solve real-world applications. In this “last mile” of implementation lie many complex challenges that may make technically high-performing systems perform poorly. Instead of viewing artificial intelligence development as a linear one of algorithm development through to eventual deployment, there are strong reasons to take a more agile approach, iteratively developing and testing artificial intelligence within the context in which it finally will be used.

Trying to separate information technology from the way it is used by people is like trying to separate a breath from the lungs that took it. We are inextricably bound to technology, and it shapes us as much as we shape it. This idea that humans and machines together constitute a larger sociotechnical system is foundational to our understanding of the modern world [1].

The sociotechnical lessons that the past hold for emerging technologies such as artificial intelligence (AI) should come as no surprise [2]. Amongst these old lessons is the maxim that the application of technology should be shaped by the problem at hand, and not the technology [3]. Although innovation in technology opens up new classes of solution, only the real world can tell us which problems are most worth solving and which solution class is most appropriate. The corollary is that a technology-driven demonstration of success with a problem does not necessarily mean it is the best solution for that problem, nor even that the problem is important.

There are thus two broad agendas for AI research [4]. The first is a technical one, pursuing new methods, architectures, and technologies that push the boundary of machine intelligence. The second is an applied agenda, where we focus on questions of implementation, extending our understanding of how best to exploit technology within the sociotechnical world it must operate.

The risk we pay for technology neophilia, our love of the new, is that we may not see the system for the technology. With AI, much research focuses on demonstrating technological success on clinical tasks such as image interpretation, diagnosis, or treatment options [5]. There are many compelling reasons for us to shift the balance from such technical demonstrations to real-world applications, not least because many technical leaps are still needed to solve challenging real-world problems.

There are three broad stages in the development of data-driven technologies like machine learning (Figure 1). Once a task such as diagnosis has been selected for algorithm development, in the “first mile,” data are acquired, possibly labelled, and preprocessed or “cleaned.” Next is the middle mile, which focusses on developing and testing the technical performance of different algorithms built using these data. Only in the last mile are algorithms embedded in real-world processes and tested for impact on real-world outcomes.

**Figure 1:**
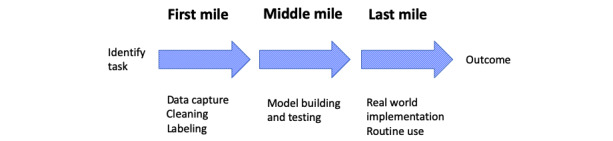
Three stages in the development of artificial intelligence technologies.

Each “mile” has its own challenges. “First mile problems” include the foundational challenges of gathering and curating high-quality data. For technologies such as machine learning, which are often dependent on large amounts of high-quality data, a bottleneck in data acquisition translates into a roadblock to technology application. The middle mile is home to all the challenges of data-driven algorithm development, including managing biases, replicability, causal inference, avoiding overfitting on training data, and enhancing the generalizability of any models and algorithms developed.

In the last mile, we face the reality that AI does not do anything on its own. It must be connecting somehow to real-world processes, and its impact on those processes needs to be consequential. It is at this point that technology developed for its own sake quickly comes to grief. For example, it is one thing to demonstrate machine learning can interpret thyroid scans for cancer as well or better than humans—a technical feat [6]; it is another for that feat to be meaningful. In the current setting, where thyroid cancer is both overdiagnosed and overtreated, we do not necessarily need better diagnoses. Instead, we need more nuanced and less aggressive approaches to management [7].

Last mile challenges are thus ones of implementation, and for researchers, of implementation science. These challenges exist at many levels, and include the following:

- *Measurement*: Standard metrics of AI performance relate to how well it completes its assigned task. We traditionally use performance measures like sensitivity, specificity, and area under the receiver operating characteristic curve (AUC). There is, however, a long chain of events that must occur between high technical performance and actual impact on clinical outcome. Success at an early stage in this information value chain is necessary, but not sufficient, to guarantee impact in the real world [8]. Evaluating real-world performance thus requires a shift from measuring technical accuracy to evaluating impact on processes and people. Measures like AUC can also be misleading in clinical settings [9]. AUC measures the overall performance of an algorithm across the entire receiver operating characteristic curve, while ideal real-world operation may best be limited to one segment of it [10].
- *Generalization and Calibration*: When an AI is trained on historic data, its future performance is dependent on how well new data match the historic data. It is a common implementation challenge to discover that a high-performing algorithm developed on data from one population deteriorates when used on a different population, reflecting underlying differences in the frequency and nature of events within the data sets. For this reason, AIs may need to be tuned to a specific end population. In many settings, this final population will also be dynamic, varying because of recurrent events like seasonal disease shifts, changes in population characteristics, and unexpected new events such as disease outbreaks. This means that AIs may need to be recalibrated, either periodically or dynamically, to reflect population changes. We also will need to closely monitor AI performance to detect shifts in its behavior that indicate that recalibration is needed [11]. More challenging is the fact that the more effectively an AI improves outcomes, the faster its performance may appear to degrade, as its very success can alter the association between predictors in its model and outcomes [12].
- *Local Context*: It is a fundamental tenet of implementation science that differences in the context in which a technology is embedded are associated with changes in performance. If we see an organization as a network of people, processes, and technologies, it is clear that the network underpinning any two organizations will be different. Implementation can be seen as the act of fitting a new technology or process into a pre-existing organizational network, and the goodness of fit of technology to network will shape any impact on organizational performance [13]. This is as true for AI as it is for digital health, in general, or for any new process or technology. To complicate matters, organizational networks are themselves dynamic. The impact of a technology will thus change with time, as the way it “fits” an organizational network changes and possibly because of its own presence—past connections will disappear or be replaced by new ones.

The software world has made a fundamental shift from seeing software development as a linear process that starts with user requirements and ends with end-user testing, to an agile one where users are embedded in a rapid and iterative process that adaptively fits software to users. Implementation science must travel the same path and especially so with dynamically changing AI.

AI development should not be seen as a linear journey stretching from the first to the last mile. Doing so risks the end product not meeting real-world needs, just as it does with software. Instead, implementation should be seen as an agile, iterative, and lightweight process of obtaining training data, developing algorithms, and crafting these into tools and workflows. Finding the right balance between reusing general technology and building to meet local needs will be crucial [14]. Either way, AI should not be created far from the place they will be used. Ideally, they should be born deep within the network that they will ultimately live in.

## Abbreviations

AI: artificial intelligence
AUC: area under the receiver operating characteristic curve
